# Tackling noncommunicable diseases in Africa through football: The case of Malawi

**DOI:** 10.1002/puh2.64

**Published:** 2023-01-28

**Authors:** Allan Kangwerema, Mictum Miggo, Gracian Harawa, Isabel Kazanga‐Chiumia, Usman Abubakar Haruna, Jackson Safari, Prosper Lutala, John Thumbiko Kaunda

**Affiliations:** ^1^ Clinical Department Nkhatabay District Hospital Nkhata Bay Malawi; ^2^ Clinical Department Queen Elizabeth Central Hospital Blantyre Malawi; ^3^ Clinical Department Mzuzu Central Hospital Mzuzu Malawi; ^4^ School of Global and Public Health, Department of Health Systems and Policy Kamuzu University of Health Sciences Blantyre Malawi; ^5^ Department of Medicine Nazarbayev University School of Medicine Astana Kazakhstan; ^6^ Department of Pharmacology and Therapeutics, Faculty of Pharmaceutical Sciences Ahmadu Bello University Zaria Nigeria; ^7^ Independent Monitoring and Evaluation Nairobi Kenya; ^8^ Department of Family Medicine Kamuzu University of Health Sciences Blantyre Malawi; ^9^ Graduate School of Health Sciences University of the Ryukyus Nishihara Japan

**Keywords:** Africa, football, health promotion, Malawi, noncommunicable diseases, sports

## Abstract

There is evidence that physical inactivity and dietary factors are more common among modifiable risk factors for noncommunicable diseases (NCDs) which are on the rise in Malawi. Football has been shown to have a wide range of health‐related benefits in health education programs, including the promotion of physical activity as a potential intervention in addressing NCDs. In Malawi, few football‐based health promotion programs have been implemented to address NCDs. Among the programs implemented were the Federation Internationale de Football Association 11 for Health initiative which was a school‐based health education program for the youth and the Malawi Ministry of Health's SPORTS FOR HEALTH initiative targeting civil servants. These programs produced significant improvement in physical activity in the participants and also increased their knowledge of both communicable and NCDs. However, these initiatives were not rolled out to many sites across Malawi. As Malawi is tackling rising cases of NCDs, football can be used as a powerful tool in promoting physical activity and it also provides an important platform for the delivery of NCDs’ health promotion messages to a wider audience. Such football‐based initiatives must be designed to directly address modifiable risk factors of NCDs. For successful implementation, there must be a good collaboration among the Ministries of Health and Sports, local football leagues, and other stakeholders.

## INTRODUCTION

Malawi, like many other Sub‐Saharan African countries, is dealing with a double burden of communicable diseases and increasing prevalence of noncommunicable diseases (NCDs) [1]. NCDs are the second leading cause of adult deaths in Malawi after HIV/AIDS accounting for 16% of all deaths with the estimated prevalence of hypertension and diabetes ranging from 15.8% to 32.9% and from 2.4% to 5.6%, respectively [1, 2]. Studies have found that physical inactivity, obesity, high salt intake, and low fruit intake were the most common modifiable risk factors for NCDs in Malawi [1]. This calls for more comprehensive and innovative ways of addressing NCDs. Regular physical activity is one of the well‐known strategies used to prevent and treat NCDs such as hypertension and diabetes mellitus.

There is increasing evidence that sporting activities have more health benefits than gym‐based physical exercises [3]. The World Health Organization (WHO) recognizes sporting activities such as football as a potential avenue where health promotion programs, including physical activity, can be delivered [4]. Given the potential benefits of sporting activities, WHO established a “sports and health program initiative” whose aim is to accelerate the attainment of the UN Sustainable Development Goal number three in ensuring healthy lives and promoting well‐being for all at all ages [4].

Football is a popular sport played by a lot of people globally as well as in Malawi. The game draws higher attendance than all other sporting events combined [3, 5]. The 2018 estimates show that 43% of the world's population identified themselves as soccer fans with male and female patronage being 63% and 37%, respectively. Of the 335 million active soccer players worldwide, 88% are male football players [6]. In Malawi, soccer is the most popular sport that is being played by young people from village teams to national teams [5]. The men's national team, the Flames, has competed in several continental competitions. There are different competitions both at the regional and national level with the Super League being the major football competition. The sport has seen increasing patronage over the years with both men and women watching different football matches in different areas both rural and urban areas [5]. In some instant, the patronage has reached over the maximum stadium capacities. This is clear evidence that football is popular in Malawi and has the power to attract many people.

The Federation Internationale de Football Association (FIFA), soccer's world governing body with 209 national soccer associations members, and WHO signed a partnership agreement in 2019 to use football to promote and safeguard the physical and mental health of people all over the world [7]. Following this partnership, several health campaigns have been launched, including the #BeActive campaign designed to encourage everyone to be physically active, and the #ReachOut campaign on mental health promotion through shared stories from football role models and FIFA legends among others [4, 7]. Through these initiatives, FIFA and WHO partnership has contributed to delivering important health education messages worldwide through campaigns and using the power of football which has resulted in the promotion of physical activity, and overall physical and mental health among soccer fans [7].

## FOOTBALL IN HEALTH PROMOTION PROGRAMS

In Malawi, football has already been utilized in different health promotion programs. One such football‐based initiative in Malawi was a series of health education programs aimed at promoting health well‐being and sexual reproductive health services among girls championed by Ascent Soccer, an organization that utilizes soccer to improve the lives of young men and women through delivery of comprehensive health education combined with soccer skill development [8]. These programs were carried out in 2021 in the three major cities of Lilongwe, Blantyre, and Mzuzu and involved football tournaments and concurrent health education awareness events on sexually transmitted infections, including HIV/AIDS [8]. Between 2018 and 2019, Malawi also carried out the Health Goals Malawi Project whose main goal was to decrease new HIV infections and sexually transmitted infections among adolescents and young people [9]. In this project, football games were used as a venue for the dissemination of health messages on HIV to young people and consequently increasing their knowledge of HIV transmission, prevention, and treatment services as well as increasing accessibility to HIV self‐testing services. [9]. These initiatives increased young people's knowledge of STI and HIV self‐testing and consequently increased the uptake of self‐testing by 20% [8, 9].

## LEVERAGING THE POWER OF FOOTBALL

As popular as it is, football has the potential to reach a wider audience and can be utilized in mass education campaigns. It can also serve as an avenue for sensitizing people to get screened for some NCDs such as hypertension and diabetes. Few initiatives have been implemented tapping into the power of football in addressing NCDs in Malawi. For example, in 2007, Malawi was among the first 5 countries in Africa where FIFA implemented a pilot project called FIFA 11 for Health which is a football‐based health education initiative aimed at increasing physical activities in children while delivering 11 simple health promotion messages to improve their health knowledge and awareness of disease prevention [10]. The program was implemented in a selected few schools and the results showed that it improved the health knowledge of communicable and NCDs among learners and increased participation and engagement in physical activity [10]. However, the initiative was not widely implemented in many public schools.

In 2015, the Ministry of Health in collaboration with the Ministry of Sports also launched an initiative called Sports for Health to raise awareness of various lifestyle‐related communicable and NCDs among public servants. This initiative included organizing sporting events, including football, to keep civil servants physically active while raising awareness of various NCDs [11]. However, the initiative increased awareness of civil servants on various communicable and NCDs to our knowledge the program was not replicated in other sites, including private and nongovernmental institutions for wide public awareness [3].

In the current NCDs national strategy, the Malawi Ministry of Health also aims at utilizing school and college physical education programs, including sporting activities such as football to foster knowledge of physical activity [2]. Although such programs have not been fully implemented in many schools, they have the potential of promoting a lifestyle that values regular physical exercises from the grassroots. Similarly, rolling out previous football‐based education projects like that of FIFA 11 to more schools across Malawi while delivering key messages on key modifiable risk factors for NCDs would be a key step in increasing awareness of NCDs and enhancing participation in physical activity [1, 10].

## FOOTBALL IN PATIENT MANAGEMENT

Recreational football has been shown to have a wide range of health benefits such as improving cardiac function, reducing blood pressure and symptoms of depression and anxiety, and improving bone and musculoskeletal health and metabolic function, among others. Football can therefore be prescribed as a broad‐spectrum non‐pharmacological treatment and prevention for NCDs [11, 12]. In Malawi, drug management forms a cornerstone of clinical care for patients with NCDs with patients being advised to engage in regular exercises as part of a prevention package [1]. In most hospitals, there are dedicated chronic care clinics that regularly attend to patients with NCDs [1].

Leveraging such already existing structures to form small teams of patients residing in proximity who can be meeting for recreational football training may have significant health benefits and consequently improve retention of exercise engagement by those patients with NCDs who often initiate the exercise but fail to continue for a long time [12, 13]. Most literature advocates pair‐based football exercises and 2 versus 2 to 5 versus 5 football training at least twice a week [12, 13]. The weekly soccer training encourages social interactions which give high rates of motivation and fulfillment, and those who engage in it are likely to continue for a long period. It also results in fewer injuries compared to match‐play football [13]. However, such a form of prescription will require the availability of local coaches who can be helping these patients in their training as well as health professionals to regularly monitor these patients.

Despite the many perceived health benefits of football, patients with limiting disabilities and the elderly population may not be able to participate in soccer due to concerns about their safety of participation in sports. As such, organizers of such football games need to actively involve the elderly in coming up with soccer programs that are tailored to their individual needs [14]. Similarly, alternatives to soccer such as walking and golf must be established to cater to those that have little or no interest in soccer.

## CONCLUSION

Football is a popular sport in Malawi among all age groups. It is a sport that forms part of physical activity which has proven non‐pharmacological benefits against NCDs, and when used as a vehicle for the delivery of health promotion messages, recreational football has the potential of spreading the messages and reaching a wider audience. As Malawi is addressing rising cases of NCDs sports activities such as recreational football is one simple and cost‐effective intervention that needs to be incorporated into NCDs treatment and prevention package. The Ministry of Health should collaborate with the Ministry of Sports, local leagues, and other stakeholders to strategically design football‐based interventions that directly address NCDs in Malawi.

## AUTHOR CONTRIBUTIONS

Allan Kangwerema, Mictum Miggo, and Gracian Harawa conceived the idea and wrote the original manuscript. Allan Kangwerema, Mictum Miggo, Gracian Harawa, Isabel Kazanga‐Chiumia, Usman Abubakar Haruna, Jackson Safari, Prosper Lutala, and John Thumbiko Kaunda contributed to reviewing and editing the manuscript. All the authors read and approved the final manuscript.

## CONFLICT OF INTEREST

One of the coauthors Isabel Kazanga‐Chiumia an Editorial Board member of Public Health Challenges and coauthor of this article. She is excluded from editorial decision‐making related to the acceptance of this article for publication in the journal.

## ETHICS STATEMENT

There is no need for ethical approval. This is a literature‐based commentary.

## Data Availability

No database or primary data was used in writing this paper.
